# The Connectivity of the Human Pulvinar: A Diffusion Tensor Imaging Tractography Study

**DOI:** 10.1155/2008/789539

**Published:** 2007-12-31

**Authors:** Sandra E. Leh, M. Mallar Chakravarty, Alain Ptito

**Affiliations:** ^1^Cognitive Neuroscience Unit, Montreal Neurological Institute and Hospital, McGill University, Montreal H3A 2B4, Canada; ^2^McConnell Brain Imaging Center, Montreal Neurological Institute and Hospital, McGill University, Montreal H3A 2B4, Canada

## Abstract

Previous studies in nonhuman primates and cats have shown that the pulvinar receives input from various cortical and subcortical areas involved in vision. Although the contribution of the pulvinar to human vision remains to be established, anatomical tracer and electrophysiological animal studies on cortico-pulvinar circuits suggest an important role of this structure in visual spatial attention, visual integration, and higher-order visual processing. Because methodological constraints limit investigations of the human pulvinar's function, its role could, up to now, only be inferred from animal studies. In the present study, we used an innovative imaging technique, Diffusion Tensor Imaging (DTI) tractography, to determine cortical and subcortical connections of the human pulvinar. We were able to reconstruct pulvinar fiber tracts and compare variability across subjects in vivo. Here we demonstrate that the human pulvinar is interconnected with subcortical structures (superior colliculus, thalamus, and caudate nucleus) as well as with cortical regions (primary visual areas (area 17), secondary visual areas (area 18, 19), visual inferotemporal areas (area 20), posterior parietal association areas (area 7), frontal eye fields and prefrontal areas). These results are consistent with the connectivity reported in animal anatomical studies.

## 1. INTRODUCTION

Previous studies in nonhuman primates and cats have shown that the pulvinar is interconnected with various subcortical and cortical areas. Neuroanatomical tracer studies demonstrated, for example, connections between the pulvinar and the amygdala, the pons, the superior colliculus, the caudate nucleus, the putamen, as well as with areas V3, V4, and V5 (MT) [1, 2]. In humans, however, the connections of the pulvinar are less well known because the number of anatomical studies is limited by methodological constraints and access only to postmortem samples.

The major retinal-cortical pathway is known to connect directly via the Lateral Geniculate Nucleus (LGN) to visual cortical areas; however, extensive corticopulvinar connections exist (e.g., [2–4]) and suggest an important role of the pulvinar in vision although its precise function remains unknown. Some studies have proposed an important role of the pulvinar in visual spatial attention, attention shifting, and visual integration [4–8] while others have suggested a contribution to sleep/wakefulness mechanisms and sensorimotor integration [9]. Electrophysiological studies in cats [6, 10, 11] as well as a recent fMRI study have further proposed an involvement of the pulvinar in higher-order visual processing [12]. With regard to the human pulvinar, its function and anatomical connections remain speculative and are based mainly on nonhuman primate research [7, 13].

The goal of the present study was to investigate human pulvinar connections in vivo. We used Diffusion Tensor Imaging (DTI) tractography, an innovative imaging technique that allows fiber tracking in vivo, to determine cortical and subcortical connections of the human pulvinar and to compare variability across normal subjects. DTI measures the random microscopic motion (diffusion) of water molecules in the brain, which allows the reconstruction of cortical fiber structures [14] by determining the diffusion direction. The preferred diffusion direction is known to be parallel to axons and can be visualized. Fiber tracing between grey matter structures can be achieved by using a probabilistic diffusion tractography algorithm and further computational analysis to reconstruct white matter fiber tracts in 3D (for further details see also [15–17] and http://www.fmrib.ox.ac.uk/fsl/fdt/index.html). Here, we demonstrate the usefulness of DTI tractography to reconstruct pulvinar tracts in humans.

## 2. METHODS

### 2.1. Subjects

Six normal subjects (3 females, 3 males), who had no history of neurological and/or ophthalmologic disorders were recruited (age range: 24–36 years). The study was approved by the Montreal Neurological Institute & Hospital (MNI) Research Ethics Committee.

### 2.2. Data acquisition

A 1.5 Tesla Siemens Sonata scanner at the Brain Imaging Center of the Montreal Neurological Institute (MNI) (Montreal, Canada) was used to obtain T_1_-weighted anatomical MRI images and diffusion-weighted images. Diffusion-weighted images were acquired by using echo-planar imaging (EPI) with a standard head coil (repetition time: 9300 milliseconds, echo time: 94 milliseconds, flip angle: 90^°^, slice thickness = 2.2 mm, number of slices: 60, in-plane resolution: 2.1875 mm × 2.1875 mm, acquisition time approximately, 9:30 minutes). Diffusion weighting was performed along 60 independent directions, with a b-value of 1000 s/mm^2^. A T_1_-weighted referenceanatomical image was also obtained also obtained.

### 2.3. Diffusion-weighted images preprocessing

Diffusion-weighted raw data were first corrected for eddy current distortions and motion artifacts [18]. We then skull-stripped the T_1_-images and fit diffusion tensors at each voxel independently to the data and coregistered diffusion-weighted images to the anatomical image using a 6-parameter transform. Diffusion modeling and probabilistic tractography were carried out using the FMRIB Diffusion Toolbox (FDT, version 1.0), which allows for an estimation of the most probable location of a pathway from a seed point using Bayesian techniques (Oxford Centre for Functional MRI of the Brain (FMRIB), FMRIB Software Library (FSL), version 5.00, UK; www.fmrib.ox.ac.uk/fsl). Fiber tracking was initiated from all voxels within the seed masks to generate 5000 streamline samples, with a steplength of 0.5 mm and a curvature threshold of 0.2. We used the FSL tools to transform anatomical images to standard space using the MNI coordinates with a 12-parameter transformation (MNI 152 brain, [19]). We first thresholded raw tracts at least at 20 samples (out of the 5000 generated from each seed voxel). We chose to use a threshold of 20 samples to remove only those voxels with very low connectivity probability. This threshold has been used previously and shown very stable results (for further details see also [20]). The results were then binarised, and summed across subject. Results are displayed as a population map, showing only reconstructed tracts that were present in at least 50% of subjects.

### 2.4. Pulvinar seed masks

**Figure 1:**
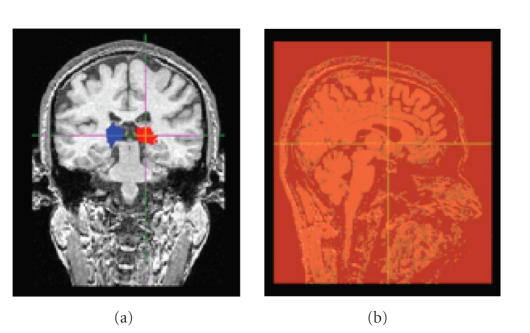
*Seedmask and exclusion mask*. Atlas was warped to a high-resolution, high-signal-to-noise ratio template. The pulvinar was extracted from the atlas and the atlas-to-subject transformation estimated for each subject was applied to the mask of the pulvinar to fit it properly to each subject. An example of the obtained right (red) and left (blue) pulvinar seedmask is shown in (a). A single sagittal slice along the midline was created on the T_1_-image of each subject to obtain an exclusion mask (b).

A digital atlas of the basal ganglia and thalamus was used [21] to create a seed mask of the left and right pulvinar on each subject's T_1_-weighted image (see Figure 1(a)). This atlas was developed from a set of high-resolution histology sliced coronally. The reconstructed data set has an in-plane voxel-to-voxel spacing of 0.034 mm while the original slice-to-slice thickness is 0.7 mm and was reconstructed using optimized nonlinear slice-by-slice morphological and intensity correction techniques.

The final atlas exists in multiple representations: The original reconstructed histological volume, a voxel-label-atlas where each structure is assigned a unique label to properly identify it, and a 3D geometric atlas. The atlas was warped onto a high-resolution, high signal-to-noise ratio template known as the colin27-MRI-average [22] using a pseudo-MRI derived from the voxel-label-atlas. The atlas-to-template nonlinear transformation was estimated using the ANIMAL algorithm [23]. The ANIMAL algorithm matches a source volume by estimating a deformation field of local translations defined on a set of equally spaced nodes which maximizes the similarity between the source and target volumes. The accuracy of this warp and the anatomical definitions on the colin27 template was compared against manual segmentations [24]. The pulvinar of each subject (target volume) was defined as a volume of interest on the atlas (source volume). A high-resolution nonlinear transformation was estimated from the atlas to fit each subject using the parameters identified in Table 1. The transformation is estimated in a hierarchical fashion where large deformations are estimated first and used as the input for the estimation of smaller, more refined transformations. All transformations are estimated on unblurred data (as an effective blurring is done in the subsampling methods used within ANIMAL). The stiffness, weight, and similarity parameters used were those identified in an optimization by Robbins et al. [25]. The final transformation is defined on a grid where local translations are grid-defined with a 1 mm isotropic spacing and then applied to the mask of the pulvinar for the DTI tractography of each subject.

### 2.5. Exclusion mask

We then created a single sagittal slice along the midline on the T_1_-image of each subject to restrict analyses to connections of one hemisphere. Fiber tracking was initiated from all voxels within the seed masks (see Figure 1).

## 3. RESULTS

A seed mask of the right and left pulvinar was created on each subject's T_1_-weighted anatomical image (see Section 2.4). Tracts were reconstructed from all voxels within the pulvinar and analysis was restricted to the ipsilateral hemisphere (see Section 2.5).

**Figure 2:**
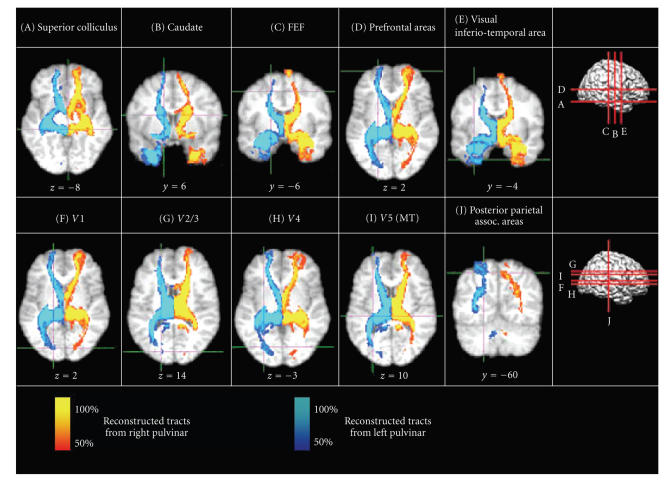
*Population probability maps of reconstructed pulvinar tracts based on tractography in six healthy subjects*. Fiber tracking was initiated from a seed mask in the right pulvinar demonstrated in red hues and from a seed mask in the left pulvinar demonstrated in blue hues. Intensity of the color scales represents the proportion of the population showing a tract at any given voxel. Tracts were registered to MNI standard stereotaxic space, thresholded at 20 samples, and binarised and summed across subjects. For individual subject tracts (see Figure 3). Images demonstrate ipsilateral connections to/from the superior colliculus (A; x = ±4, y = −34, z = −8), the caudate (B; x = ±12, y = 6, z = 16), the frontal eye fields (C; x = ±18, y = −6, z = 50), prefrontal areas (D; x = ±20, y = 62, z = 2), visual inferiotemporal area (E; x = ±32, y = −4, z = −42), V1 (F; x = ±16, y = −86, z = 2), V2/3 (G; x = ±16, y = −88, z = 14; x = ±22, y = −80, z = 22), V4 (H; x = ±26, −75, −3), V5 (MT) (I; x = ±32, y = −74, z = 10), and posterior parietal association areas (J; x = ±20, y = −60, z = 54). Note the high consistency of pulvinar tracts across subjects.

**Figure 3:**
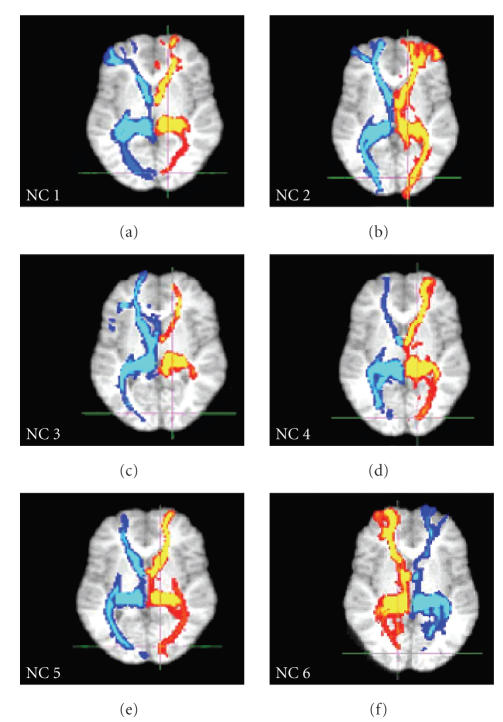
*Examples of individual pulvinar in six control subjects (NC1-6)*. Tracts (slice level V1: x = 16, y = −86, z = 2) have been thresholded at 20 samples. Red hues demonstrate reconstructed tracts from the right pulvinar, and blue hues demonstrated reconstructed tracts from the left pulvinar. The intensity of color scales indicates the number of samples that passed through that voxel from red/blue (low probability of connection) to yellow/light blue (high probability of connection).

Reconstructed pulvinar tracts are displayed as a population map in Figure 2. Only tracts that were present in at least 50% of the subjects are shown. Examples of individual pulvinar tracts are demonstrated in Figure 3. Reconstructed tracts of the right and left pulvinar projected ipsilaterally to the superior colliculus (A; x = ±4, y = −34, z = −8), the caudate (B; x = ±12, y = 6, z = 16), the frontal eye fields (C; x = ±18, y = −6, z = 50), prefrontal areas (D; x = ±20, y = 62, z = 2), visual inferotemporal area (E; x = ±32, y = −4, z = −42), V1 (F; x = ±16, y = −86, z = 2), V2/3 (G; x = ±16, y = −88, z = 14; x = ±22, y = −80, z = 22), V4 (H; x = ±26, −75, −3), V5 (MT) (I; x = ±32, y = −74, z = 10), and posterior parietal association areas (J; x = ±20, y = −60, z = 54). Please note the high consistency of pulvinar tracts across subjects.

## 4. DISCUSSION

Our DTI tractography study in humans reveals connections between the pulvinar and various cortical and subcortical areas. In accordance with previous primate studies, connections to the superior colliculus and caudate were observed [1–3, 26–29]. Our reconstructed pulvinar tracts also projected ipsilaterally to prefrontal, inferior temporal, and parietal association areas, as previously shown in nonhuman tracer studies [27, 30–32]. Furthermore, we were able to confirm the existence of connections between the human pulvinar and visual cortical areas V1, V2, V4, and V5 (MT) in keeping with studies in nonhuman primates [2, 8, 33]. Finally, we found that connections between the pulvinar and the frontal eye fields that were previously shown to exist in monkeys [34–36] are also present in humans.

Taken together, our study demonstrates that extensive cortical and subcortical connections from/to the pulvinar also exist in humans. Although DTI tractography is not able to distinguish between feedforward and feedbackward projections, our reconstructed tracts suggest that the pulvinar likely plays an important role in human visual information processing and visual spatial attention as it does in nonhuman primates and cats [4, 6, 12]. Our results also demonstrate that DTI tractography is a useful new imaging technique to investigate human anatomical pathways.

## Figures and Tables

**Table 1:** Atlas-to-subject warping parameters used to estimate a high-resolution nonlinear transformation using the ANIMAL algorithm.

Step	Step size (mm)	Sub-lattice diameter (mm)	Sublattice	Iterations
1	4	8	8	15
2	2	6	8	15
3	1	6	6	15
